# Unequal Regional Access to ACL Reconstruction in Romania: A Nationwide Epidemiologic Assessment (2017–2023)

**DOI:** 10.3390/medicina62010193

**Published:** 2026-01-16

**Authors:** Gloria Alexandra Tolan, Cris Virgiliu Precup, Roxana Furau, Bogdan Uivaraseanu, Delia Mirela Tit, Gabriela S. Bungau, Mirela Marioara Toma, Tiberiu Sebastian Nemeth, Cristian George Furau

**Affiliations:** 1 Multidisciplinary Doctoral School, “Vasile Goldis” Western University of Arad, 310414 Arad, Romania; gloria.tolan@yahoo.de (G.A.T.); furau.cristian@uvvg.ro (C.G.F.); 2 Department of Life Sciences, “Vasile Goldis” Western University of Arad, 310414 Arad, Romania; precupcris@yahoo.com; 3 Department of General Medicine, Faculty of Medicine, “Vasile Goldis” Western University of Arad, 310414 Arad, Romania; furau.roxana@uvvg.ro; 4 Department of Surgical Disciplines, Faculty of Medicine and Pharmacy, University of Oradea, 410073 Oradea, Romania; 5 Doctoral School of Biological and Biomedical Sciences, University of Oradea, 410087 Oradea, Romania; gbungau@uoradea.ro (G.S.B.); snemeth@uoradea.ro (T.S.N.); 6 Department of Pharmacy, Faculty of Medicine and Pharmacy, University of Oradea, 410028 Oradea, Romania; toma.mirelamarioara@didactic.uoradea.ro; 7 Department of Pathophysiology, Faculty of Medicine, “Vasile Goldis” Western University of Arad, 310414 Arad, Romania

**Keywords:** anterior cruciate ligament reconstruction, incidence, epidemiology, geographic variation, socioeconomic determinants, surgical workforce, Romania

## Abstract

*Background and Objectives*: Access to anterior cruciate ligament reconstruction (ACLR) varies substantially across health systems, yet national-level data from Eastern Europe remain limited. This study provides the first nationwide, regionally stratified assessment of ACLR activity in Romania, examining geographic variation, socioeconomic and workforce determinants, and inequality. *Materials and Methods*: We conducted a retrospective cross-sectional analysis of all ACLRs reported in the national administrative hospital database (2017–2023), supplemented with demographic, GDP, and workforce statistics. Outomes included incidence per 100,000 population, private-sector share, and sex distribution. Regional differences were tested using Kruskal–Wallis and Dunn post hoc comparisons. Predictors of ACLR incidence and private-sector utilization were identified through multivariable Poisson and logistic models. Inequality metrics (Gini coefficients, P90/P10 ratios) and sensitivity analyses excluding Bucharest–Ilfov were also performed. *Results*: A total of 11, 080 ACLRs were recorded. Incidence varied markedly across regions, from a median of 40.0 per 100,000 in Bucharest–Ilfov to <1–3 per 100,000 in the South, South-East, and South-West (*p* < 0.001). Higher GDP per capita correlated with incidence (ρ = 0.36) and explained 45% of its variance. Private-sector involvement ranged from <5% in Bucharest–Ilfov and the South to 80–100% in the Centre, North-West, and South-East. In adjusted Poisson models, GDP, surgeon availability, and private-sector share were strong independent predictors of incidence (all *p* < 0.001). Private-sector access was primarily determined by the proportion of private orthopedic surgeons (OR 21.03). National inequality was extreme (Gini 0.842–0.752; P90/P10 > 10^9^), reflecting the concentration of procedures within a small number of counties. Results were consistent across sensitivity analyses. *Conclusions*: ACLR in Romania displays severe territorial inequities driven by socioeconomic development, workforce distribution, and uneven private-sector capacity. Targeted regional investment and coordinated workforce strategies are necessary to improve equitable access to surgical care.

## 1. Introduction

Anterior cruciate ligament (ACL) injury remains one of the most frequent and functionally debilitating knee injuries, particularly among young and physically active individuals. It often leads to knee instability, impaired proprioception, and substantial limitations in athletic and occupational performance [1,2]. Beyond the immediate impact, untreated or inadequately managed ACL rupture predisposes patients to secondary meniscal and chondral lesions, chronic pain, and early-onset osteoarthritis, with long-term consequences for joint health and quality of life [3,4]. For individuals seeking to return to pivoting or high-demand activities, ACL reconstruction (ACLR) is widely recognized as the gold-standard surgical approach for restoring mechanical stability and preventing further intra-articular deterioration [5,6].

In recent decades, ACL reconstruction techniques have evolved considerably. Refinements in graft options, tunnel placement accuracy, fixation methods, and arthroscopic visualization have contributed to improved anatomical restoration and more predictable surgical outcomes [7,8,9]. Concurrent advances in diagnostic imaging, perioperative pain management, and accelerated rehabilitation protocols have facilitated earlier mobilization and higher rates of return to sport. Despite these technical improvements, ACLR incidence continues to rise globally, driven by increased participation in organized sports, enhanced diagnostic availability, and expanded orthopedic capacity [10,11].

However, multiple studies have demonstrated that the utilization of ACL reconstruction varies considerably both between and within countries [12,13,14]. Nordic nations such as Sweden, Norway, Denmark, and Finland consistently report some of the highest ACLR rates worldwide, whereas Southern European countries demonstrate substantially lower utilization. For example, national registry data from Finland reported an overall ACLR incidence of 46 per 100,000 person-years, with mid-study peaks exceeding 50 and marked sex differences, as men consistently exhibited higher rates than women [14]. In Norway, long-term registry analyses revealed a substantial increase in pediatric and adolescent ACLR—rising by 40% in boys and 55% in girls over a 16-year period—alongside evolving surgical practices, including a shift from meniscal resection to repair [15]. In contrast, the Italian nationwide registry demonstrated lower ACLR incidence (approximately 22–34 per 100,000 inhabitants) and pronounced territorial asymmetry, with more than two-thirds of procedures clustered in northern regions despite comparable population sizes across the country [16].

These discrepancies are not attributable solely to differences in sports participation or injury epidemiology but also reflect underlying structural and socioeconomic determinants of healthcare access. Health system investment, the distribution of specialized surgeons, hospital infrastructure, and referral pathways have all been identified as key contributors to geographic variation in orthopedic surgical care [17,18,19]. Eastern European countries remain notably underrepresented in ACL epidemiology and service-delivery research, despite well-documented inequalities in healthcare infrastructure, specialist workforce allocation, and socioeconomic development [20].

Romania exemplifies this pattern, as national reviews describe persistent challenges related to healthcare financing, workforce shortages, and unequal territorial distribution of services, with a strong urban–rural contrast in access to care [21]. These structural features are associated with uneven availability of hospital infrastructure and specialist services across the country and may influence access to elective orthopedic pathways [21].

In line with this broader context, a nationwide register-based analysis of orthopedic surgical activity in Romania reported substantial county-level heterogeneity in procedure volumes and per-capita densities, including very high values in major university medical centers and very low densities in several counties [22]. This territorial variation was observed across multiple elective orthopedic domains captured in the national registry, including arthroplasty and arthroscopic procedures, underscoring the role of hospital distribution and workforce availability in shaping procedural access [22]. Our previous national analysis of arthroscopic knee procedures [23] revealed steadily rising ACLR volumes across Romania between 2017 and 2023, reflecting growing procedural demand and evolving surgical practice patterns. Building upon these findings, the present study investigates the structural and socioeconomic determinants of ACL reconstruction, providing the first nationwide, regionally stratified analysis of surgical activity and inequality in Romania. By linking administrative data with workforce and economic indicators, this research aims to clarify how system-level contextual factors shape access to reconstructive knee surgery. Understanding these mechanisms is essential for promoting equitable healthcare delivery, optimizing surgical resource allocation, and supporting evidence-based policy decisions to improve musculoskeletal outcomes nationwide.

## 2. Materials and Methods

### 2.1. Study Design and Data Sources

We conducted a retrospective, cross-sectional study using secondary analysis of national administrative data for all anterior cruciate ligament reconstructions (ACLR) performed in Romania between 1 January 2017 and 31 December 2023. The primary data source was the Romanian National Health Insurance House (CNAS), which compiles mandatory hospitalization records from all public hospitals and from private hospitals contracted with the national insurance system under Ministry of Health regulations. From the national surgical database, we extracted all procedures coded as O15303, which represents arthroscopic anterior cruciate ligament reconstruction in the NCSP classification. This is the single, nationally used NCSP code that uniquely identifies ACLR in Romania, providing complete specificity for the detection of primary ACL reconstruction procedures. No other knee arthroscopy codes (e.g., meniscectomy, synovectomy, or unspecified ligament reconstruction procedures) were included, ensuring that only true ACL reconstruction cases were captured.

For each procedure, the CNAS dataset provided the hospital identifier, county, year of intervention, sector (public/private), patient sex, and age group. The database contains all reimbursed inpatient interventions; procedures performed exclusively in fully out-of-pocket private clinics, non-contracted with CNAS, are not captured. As such, the dataset reflects the complete population of ACL reconstructions performed within the national insurance system, while likely underestimating the true national volume by omitting a small number of privately self-paid cases.

Access to both the surgical procedures dataset and county-level orthopedic workforce data was granted by the National Institute of Public Health (INSP) under formal authorization no. 20606/5 December 2024 and no. 21269/21 November 2025, permitting use of anonymized administrative records for research purposes. The surgical workforce variable represented the total number of orthopedic surgeons per county, as reported by the National Institute of Public Health. Data on sub-specialization or specific procedural practice (e.g., arthroscopic or sports medicine surgeons) were not available at the national level. Consequently, this indicator captures overall orthopedic workforce availability rather than the subset directly performing ACL reconstruction. It was selected as the most comprehensive and consistent measure of surgical capacity across regions.

Data processing and analysis were performed by a multidisciplinary team of researchers and clinicians with doctoral-level training and prior experience in orthopedic surgery, epidemiology, and quantitative data analysis. As this was a retrospective, population-based analysis including all ACL reconstructions recorded in the national database, no randomization or sampling procedures were applied.

**Figure 1:**
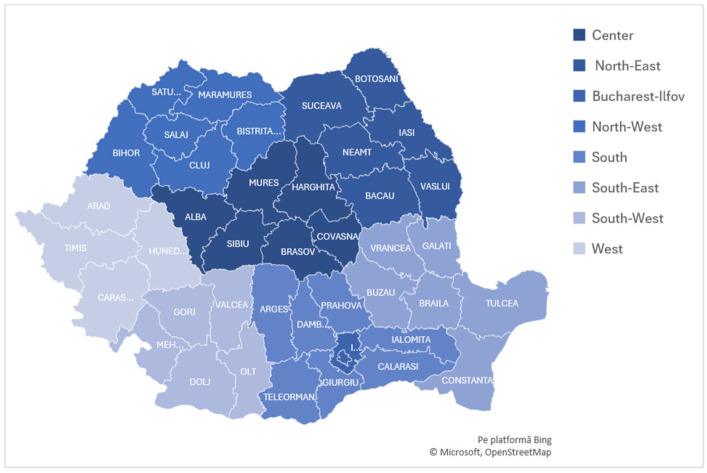
Map of Romania showing the eight development regions (NUTS-2) and their component counties, used as the geographic units for all regional analyses in this study.

Spatial analyses were conducted using the eight Romanian development regions, defined according to the Nomenclature of Territorial Units for Statistics (NUTS-2), which represents the standard territorial framework for health-system and socioeconomic comparisons [24]. Figure 1 provides a schematic map of these regions and their component counties, offering geographic context for interpreting regional variation in incidence, workforce availability, and structural indicators.

County-level population counts were sourced from the 2021 Romanian Population and Housing Census (INSSE) [25]. To characterize regional socioeconomic context, we used county-level gross domestic product (GDP) per capita from the same reference year, obtained from the National Institute of Statistics (INSSE) and reported in Romanian lei [26]. GDP values were converted to euros using the official annual exchange rate (4.92 RON/EUR), ensuring consistency across demographic and economic indicators without introducing temporal misalignment.

Orthopedic workforce counts were supplied directly by INSP and represent the official registry of licensed orthopedic surgeons practicing in each county during the study period. Hospital availability was extracted from the CNAS dataset and reflects only facilities reporting at least one ACL reconstruction, thereby quantifying the functional surgical network capable of delivering ACLR within each region.

### 2.2. Outcome Measures and Statistical Analysis

The primary outcome was the incidence of ACL reconstruction (ACLR) per 100,000 inhabitants at NUTS-2 regional level. Incidence was calculated using the standard epidemiological formula, in which the annual number of ACLRs performed in a given region was divided by the census-based 2021 regional population and multiplied by 100,000.

Secondary outcomes included the proportion of ACLRs performed in the private sector, the male-to-female sex ratio, regional surgeon density (orthopedic surgeons per 100,000 inhabitants), and hospital availability (ACL-performing hospitals per 100,000 inhabitants). Annual workload was also assessed by calculating the number of ACLRs performed in each region relative to the number of practicing orthopedic surgeons. These measures were used to characterize regional differences in service capacity, structural resources, and potential determinants of inequality in access to ACL reconstruction.

All analyses were performed using JASP v0.19.3 and Microsoft Excel. Continuous variables were summarized using medians, means, standard deviations, ranges, and interquartile ranges (IQR). Because most distributions were non-normal (Shapiro–Wilk, *p* < 0.05), regional comparisons of incidence were performed using the Kruskal–Wallis test, followed by Dunn post hoc comparisons with Holm correction. Effect sizes were reported using rank epsilon-squared (ε^2^).

To examine structural determinants of access, we fitted multivariable regression models, all weighted by county population:

1. Poisson regression predicting ACL incidence (per 100,000), including year, GDP per capita (*z*-scored), proportion of private-sector ACLRs, total orthopedic surgeons, and NUTS-2 region. Sensitivity analyses were conducted by excluding Bucharest–Ilfov, given its disproportionately high volume.

To further evaluate temporal variation in regional inequalities, an extended Poisson model was also estimated including *Year* × *Region* interaction terms, adjusted for the same covariates

2. Workforce-adjusted Poisson regression, using log(total surgeons) as an offset, estimating surgical access per available surgeon.
3. Binomial logistic regression predicting the probability of receiving ACLR in a private hospital.
4. A sex-based logistic regression was also estimated to evaluate demographic imbalances. All models used robust standard errors.

Robust standard errors were used for all models. GDP per capita was standardized using *z*-scores to ensure comparability across variables.

National and intra-regional inequality in ACLR distribution were quantified using two complementary measures, Gini coefficient and P90/P10 extreme ratio [27]. The Gini coefficient was calculated using the standard discrete formula based on pairwise absolute differences:

$$G=\Sigma_{i}\Sigma_{j}\;|x_{i}-x_{j}|/(2\cdot n^{2}\cdot \bar{x})$$

where $x_{i}$ and $x_{j}$ denote county-level ACL incidence values, n  represents the number of counties, and $\bar{x}\;$ is the national mean incidence. This formulation captures the normalized average disparity between all possible pairs of counties.

To evaluate tail-end disparity, we computed the ratio of the 90th to the 10th percentile of county-level incidence:

P90/P10 = P_90_(*x*)/P_10_(*x*)

Because several counties recorded near-zero ACLR activity, the denominator approached zero, generating ratios on the order of 10^9^–10^10^, consistent with extreme geographic polarization.

### 2.3. Validation and Robustness Analyses

Robustness analyses were conducted to ensure that the observed associations and inequality estimates were not driven by regional outliers, incomplete reporting, or modeling assumptions.

To test the stability of regional predictors, all multivariable models were re-estimated after excluding Bucharest–Ilfov, the region with the highest ACLR volume. Alternative model specifications were also tested, including using workforce-adjusted incidence (log surgeons as offset), robust standard errors to address potential overdispersion, and *Year* × *Region* interactions to evaluate temporal consistency.

To further evaluate the robustness of inequality metrics, we conducted a dedicated sensitivity analysis simulating unreported ACLRs performed in fully private, out-of-pocket (OOP) settings. This simulation used empirically informed assumptions based on observed surgical workload distributions. County-level data on ACLRs per surgeon were used to estimate typical annual workloads. Since only a minority of surgeons performed more than five ACLRs per year, we conservatively assumed that only a subset of private-sector surgeons was actively contributing to ACLR volume.

For each county, the number of potentially active private surgeons was estimated by multiplying the total number of orthopedic surgeons by the reported local proportion working in the private sector. To avoid overestimation, the simulated case volume was constrained by plausible workload limits based on observed activity.

Three workload scenarios were applied: 5, 10, and 15 ACLRs per active private surgeon per year. Simulated cases were proportionally distributed across counties, added to observed counts, and used to recalculate adjusted incidence rates, Gini coefficients, and P90/P10 ratios.

This approach allowed us to evaluate whether unreported procedures in the private sector could meaningfully alter national or regional inequality metrics, and whether the observed disparities represent conservative or inflated estimates.

## 3. Results

### 3.1. Descriptive Characteristics and Regional Variation

Between 2017 and 2023, a total of 11,080 ACL reconstructions were recorded nationwide. Consistent with known epidemiology, procedures were predominantly performed in males, with a stable male-to-female ratio of approximately 2–3:1 across all regions, without a detectable geographic pattern. Marked regional heterogeneity was observed in crude incidence rates (per 100,000 inhabitants). Bucharest–Ilfov exhibited by far the highest values, with a median of 40.0/100,000, exceeding all other NUTS-2 regions across the entire study period. Intermediate levels were observed in Centre, West, and North-West, whereas South, South-East, and especially *South*-*West* consistently recorded the lowest incidence values (Table 1).

**Figure 2:**
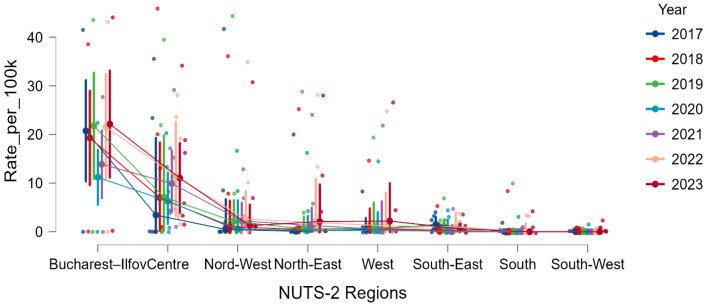
Annual ACL reconstruction incidence across NUTS-2 regions in Romania (2017–2023). Each point represents a county-year observation; lines connect annual regional medians.

Figure 2 visualizes the annual incidence trajectories, highlighting a highly asymmetric geographic distribution that remained stable over time despite moderate year-to-year variation. Bucharest–Ilfov persistently dominates the national profile, whereas all southern regions cluster at the lower end throughout the seven-year period.

A Kruskal–Wallis test confirmed significant regional variation in ACL reconstruction rates (H(7) = 46.77, *p* < 0.001), with a large effect size (rank ε^2^ = 0.29; 95% CI: 0.22–0.43). Post hoc Dunn tests (Holm-corrected) indicated that Bucharest–Ilfov had significantly higher rates than North-East, South, South-East, and South-West, and partially higher than North-West (adjusted *p* < 0.05), but did not significantly differ from Centre or West (Table 2). Similarly, the Centre region recorded significantly higher rates than North-East, South, South-East, and South-West.

Together, these findings confirm a clear and persistent geographic gradient, spanning high-incidence (Bucharest–Ilfov, Centre), intermediate (West, North-West), and consistently low-incidence regions (South, South-East, South-West).

Substantial regional variability was also present in the public–private distribution of ACL reconstructions (Table 3). Bucharest–Ilfov had the lowest private-sector share (median 3.3%), with most cases performed in public hospitals despite the region’s high surgical volume. In contrast, Centre, North-West, South-East, and West exhibited substantial private-sector involvement, occasionally exceeding 80–100% in specific years. The South-West region recorded zero private ACL reconstructions during the entire study period.

**Figure 3:**
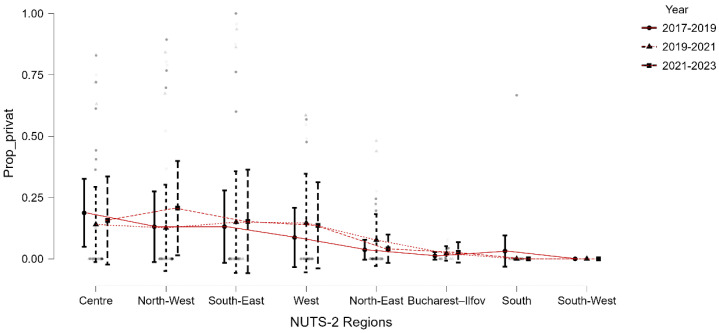
Proportion of ACL reconstructions performed in private hospitals across NUTS-2 regions, grouped by three time periods (2017–2019, 2019–2021, 2021–2023). Error bars represent within-region variability.

Temporal patterns displayed a modest post-pandemic increase in private-sector utilization (2021–2023), particularly in Centre, North-West, and South-East. However, this trend was absent in South and South-West, which remained almost entirely dependent on the public system. Figure 3 illustrates these trends across the three aggregated periods, demonstrating a stable geographic gradient in private-sector involvement and a partial post-pandemic expansion of private surgical capacity.

### 3.2. Socioeconomic Determinants and Healthcare Capacity

Regional economic development showed substantial heterogeneity across Romania, with clear gradients reflected in both healthcare workforce availability and ACL reconstruction activity. After conversion from RON to euro, Bucharest–Ilfov remained the wealthiest region (median GDP per capita €23,724), followed by the West (€10,669) and North-West (€9543), whereas the North-East showed the lowest economic levels (median €7474). Summary statistics for all structural indicators (GDP per capita, surgeons per 100,000 inhabitants, hospitals per 100,000 inhabitants, and cases per surgeon) are presented in Table 4.

Higher economic development was consistently associated with increased orthopedic workforce density and greater surgical activity. Regions with elevated GDP per capita showed higher numbers of surgeons per 100,000 inhabitants and more available hospital infrastructure, while lower-GDP regions displayed sparse workforce distribution and limited surgical capacity. Bucharest–Ilfov had nearly double the surgeon density of most other regions and more than fourfold the ACL case load per surgeon compared with the lowest performing areas.

**Figure 4:**
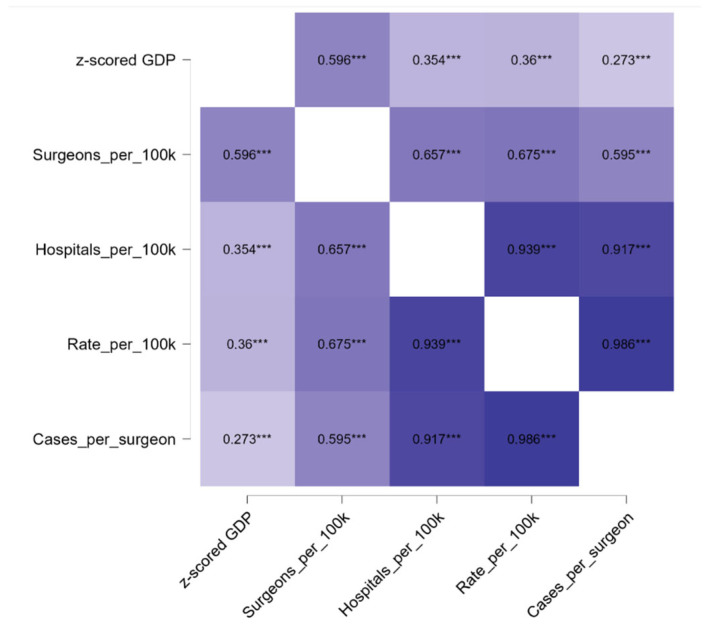
Correlation matrix between regional socioeconomic indicators, healthcare capacity, and ACL reconstruction utilization. GDP per capita was standardized using *z*-scores to allow comparability across regions. Darker shading indicates stronger correlations; *** *p* < 0.001.

The correlation heatmap (Figure 4) illustrates these structural alignments. GDP per capita demonstrated moderate correlations with surgeon density (r = 0.596, *p* < 0.001), hospital availability (r = 0.354, *p* < 0.001), and ACL incidence (r = 0.360, *p* < 0.001). Strong associations were observed within the healthcare system: surgeons per 100 k strongly correlated with hospitals per 100 k (r = 0.657, *p* < 0.001) and with surgeon-level workload (cases per surgeon; r = 0.595, *p* < 0.001), while hospital density was closely linked to cases per surgeon (r = 0.917, *p* < 0.001).

These findings show that regional economic strength aligns robustly with healthcare capacity and ACL reconstruction activity.

### 3.3. Multivariate Models: Adjusted Predictors of ACL Reconstruction Access

#### 3.3.1. Poisson Regression Model Predicting ACL Reconstruction Incidence

The multivariate Poisson model demonstrated substantial and highly significant regional inequalities in ACL reconstruction (Table 5). After full adjustment for GDP per capita, surgeon workforce, year, and private-sector share, region remained the strongest independent predictor, with incidence markedly higher in Bucharest–Ilfov and substantially lower across all other NUTS-2 regions.

Higher socioeconomic status and specialist availability were major determinants of increased access. GDP per capita was positively associated with incidence (IRR = 3.99, 95% CI: 2.39–6.66, *p* < 0.001), total orthopedic surgeons had a modest but significant effect (IRR = 1.01, *p* < 0.001) and year effects reflected the known pandemic disruption, with sharp declines in 2020–2021 and recovery afterward. The share of private-sector procedures did not independently predict incidence (*p* = 0.29), suggesting that private capacity influences where procedures occur but not the overall volume within regions.

To evaluate stability, the Poisson model was re-estimated excluding Bucharest–Ilfov.

Predictor effects remained almost identical in magnitude and significance, with GDP (IRR = 1.96), private share (IRR = 1.60), and surgeon supply (IRR = 1.02) all robustly associated with incidence. Regional inequalities persisted, with South-West and South-East showing the lowest IRRs (0.05–0.14). This confirms that findings are not driven by volume concentration in the capital.

To further assess whether the regional inequalities identified in the main Poisson model changed over time, an additional model including *Year* × *Region* interaction terms was estimated, adjusted for the same covariates (GDP per capita, orthopedic surgeon supply, and private-sector share). The interaction model revealed significant temporal heterogeneity in ACLR incidence across regions (χ^2^ = 11,625.7, *p* < 0.001). Several regions exhibited marked divergence during the pandemic period (2020–2021), with steep temporary declines in Bucharest–Ilfov and partial rebounds in economically stronger regions such as Centre, West, and North-West. In contrast, low-GDP regions (South, South-East, South-West) showed delayed or minimal recovery in post-pandemic years (2022–2023), indicating persistence or even widening of access disparities.

Interaction coefficients confirmed significant year-dependent shifts: for example, the *North*-*East* × 2021 (IRR = 6.68, 95% CI 5.14–8.64, *p* < 0.001) and *West* × 2021 (IRR = 5.54, 95% CI 3.97–7.81, *p* < 0.001) terms reflected strong regional rebounds following the 2020 collapse, while southern regions displayed weaker or inconsistent trends. The model suggests that the pandemic amplified pre-existing geographic inequalities, with slower recovery among resource-poor regions despite national normalization of surgical volumes. Detailed interaction effects are reported in Table 6, showing incidence rate ratios (IRRs) for each region-year combination relative to Bucharest–Ilfov in 2017 (reference).

**Figure 5:**
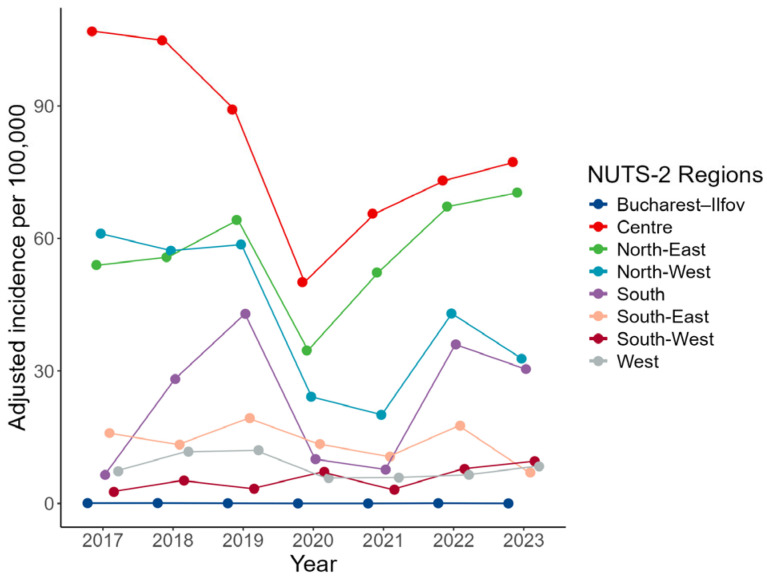
Predicted ACL reconstruction incidence by year and NUTS-2 region, estimated from a Poisson regression including a *Year* × *Region* interaction and adjusted for GDP per capita, surgeon density, and private-sector share.

Adjusted predictions (Figure 5) demonstrate that incidence in the Centre and North-East regions rebounded sharply post-2021, while the South and South-West remained persistently low.

Bucharest–Ilfov served as the reference category; its values are displayed on the model’s intercept scale.

#### 3.3.2. Workforce-Adjusted Model: Surgical Capacity as a Determinant of Access

To examine whether regional disparities in ACL reconstruction are driven by underlying differences in orthopedic surgical capacity, we estimated a workforce-adjusted Poisson model using total ACL cases as the dependent variable and log (Total surgeons) as an offset. This specification standardizes ACL volume per available surgeon, allowing assessment of structural factors independent of workforce size.

The model showed excellent fit (Deviance = 2552.2, df = 151, *p* < 0.001). GDP per capita remained a strong, independent predictor of higher ACL reconstruction volume (IRR = 1.78 per SD increase in GDP; *p* < 0.001). A small but significant year-to-year reduction was retained (IRR = 0.97, *p* < 0.001), consistent with national trends showing incomplete post-COVID recovery. Even after workforce normalization, pronounced regional disparities persisted. Compared with Bucharest–Ilfov, all regions except South-West showed significantly higher ACL incidence per available surgeon, with IRRs ranging from 4.5 (West) to over 21 (North-East) (Table 7). This indicates that disparities are not solely explained by specialist concentration but reflect broader structural and socioeconomic differences in surgical access.

Overall, the workforce-adjusted model confirms that surgical capacity contributes to, but does not eliminate, regional variation in ACL reconstruction, supporting a dual mechanism driven by both workforce distribution and socioeconomic environment.

#### 3.3.3. Predictors of Private-Sector ACL Reconstruction (Binomial Logistic Model)

The multivariable logistic regression model showed that private-sector supply factors overwhelmingly determine the likelihood of undergoing ACL reconstruction in a private hospital (Table 8). The strongest predictor was the proportion of private orthopedic surgeons (OR = 21.03, *p* < 0.001), indicating exponential increases in private-sector utilization when private workforce availability is high.

Higher GDP also increased the odds of private intervention (OR = 1.58, *p* < 0.001), while total surgeon supply exerted a smaller but significant positive effect (OR = 1.01, *p* < 0.001). A slight decline in private sector use over time was observed (OR = 0.97, *p* = 0.044).

Regional disparities were extreme: all regions except South-West showed massively increased odds of private-sector reconstruction relative to Bucharest–Ilfov, with ORs ranging from 766 (South) to >60,000 (South-East), reflecting very low private-sector penetration in the capital compared with other parts of the country.

#### 3.3.4. Sex-Based Model (Male vs. Female Access)

The sex-based logistic regression model demonstrated minimal evidence of sex-related differences in access to ACL reconstruction (Table 9). GDP, surgeon supply, and year showed no association with the likelihood of a patient being male. Regional differences were also largely absent, except for the North-East, where cases had twice the odds of being male compared with Bucharest–Ilfov (OR = 2.00, *p* = 0.033). This indicates that observed inequities in ACL reconstruction access are structural and socioeconomic, not driven by demographic imbalance between male and female patients.

Across all analytical models, three structural determinants consistently emerged as the strongest predictors of access to ACL reconstruction in Romania: the regional socioeconomic context reflected by GDP per capita, the distribution of the orthopedic surgical workforce, and the availability of services in the private sector. These system-level factors dominated the explanatory landscape, overshadowing individual-level characteristics. In contrast, sex-based disparities were negligible.

### 3.4. Inequality Metrics

**Figure 6:**
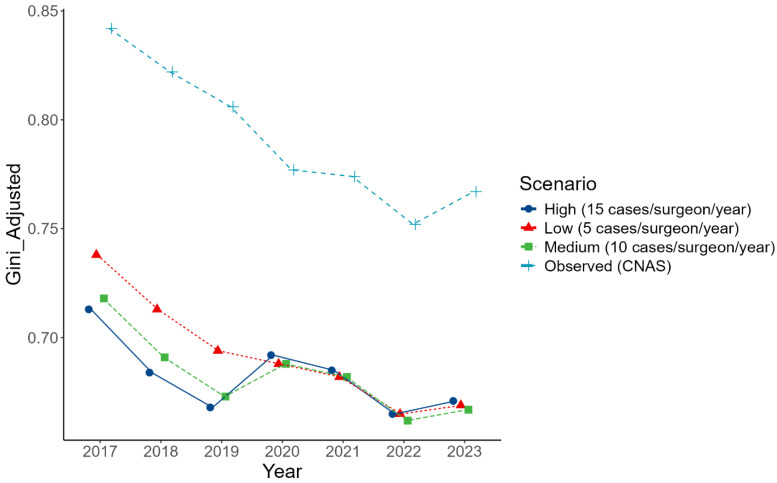
Annual Gini coefficient for ACLR incidence by county under observed and simulated private-sector scenarios (2017–2023).

Substantial geographic inequality was identified in the national distribution of ACL reconstruction incidence. The national Gini coefficient ranged from 0.842 in 2017 to 0.752 in 2022, indicating extreme and persistent territorial concentration of surgical activity. Although a modest decline was observed after the COVID-19 peak, values remained far above the conventional threshold for severe imbalance (Gini > 0.5), confirming enduring structural disparities. To quantify the possible effect of underreported private-sector activity, particularly fully out-of-pocket procedures not captured by the national database, we performed a three-scenario sensitivity analysis, redistributing simulated cases to counties with non-contractual private surgical capacity (see Methods). The adjusted Gini coefficients declined markedly across all years, reaching 0.713 (2017) in the high-case scenario (15 cases per private-sector surgeon/year), 0.718 in the medium-case, and 0.738 in the low-case (5 cases/surgeon/year). Similar reductions were observed in subsequent years, with the adjusted Gini falling to as low as 0.665 in 2022, depending on the scenario. Even under high-case assumptions, national inequality metrics remain substantially elevated (Table 10 and Figure 6)

The P90/P10 incidence ratio further highlighted the magnitude of inequality, reaching between 10^9^ and 10^10^ across all years. This reflects the fact that counties in the lowest decile recorded near-zero ACL activity, while those in the highest decile concentrated almost the entire national surgical volume. Such divergence is consistent with highly polarized access to specialized orthopedic care.

Inequality was also pronounced within development regions (NUTS-2) (Table 11). Internal Gini coefficients ranged from 0.555 in Bucharest–Ilfov to 0.867 in the South region, with Nord-Est, Sud-Vest, Vest, and Nord-Vest all exceeding 0.73. Adjusted intraregional Gini values remained high in the South even after simulation (0.740–0.747), confirming persistent polarization within this under-resourced area

Thus, disparities are not only inter-regional but also deeply embedded within regions themselves, indicating strong concentration of surgical capacity in a minority of counties even inside the same NUTS-2 area.

## 4. Discussion

This study provides the first nationwide, regionally stratified evaluation of anterior cruciate ligament reconstruction (ACLR) activity in Romania, integrating administrative surgical data with socioeconomic and workforce indicators. Our findings reveal substantial and persistent territorial variation in ACLR rates, shaped primarily by differences in healthcare capacity and regional economic development rather than demographic characteristics. Similar patterns have been described internationally, where variations in service provision frequently reflect structural differences in health system resources, specialist availability, and local infrastructure rather than true differences in clinical need [28,29,30].

### 4.1. Regional and Structural Inequalities in ACL Reconstruction

A marked geographic imbalance was evident across Romania. The Bucharest–Ilfov region consistently demonstrated the highest ACLR incidence, approaching levels observed in high-income healthcare systems, while several regions, particularly in the South, South-East, and South-West, reported extremely low rates. The stability of this gradient over the entire 2017–2023 period suggests a structural access pattern rather than short-term fluctuation. The absence of a meaningful geographic pattern in the male-to-female ratio further indicates that regional differences in utilization are unlikely to be driven by demographic composition, and more likely reflect service availability, referral pathways, and surgical capacity concentration.

Geographic variation in access to medical and surgical services is a well-documented phenomenon, even among countries with advanced health systems, and is often attributed to differences in service supply, workforce distribution, and regional socioeconomic context [30,31,32]. Comparable disparities have been documented across Europe. For instance, in Italy, ACLR rates are highest in wealthier northern regions, while southern and rural areas lag behind despite comparable population needs [16]. Thus, the inequalities observed in Romania are not unique but rather reflect a continuum of structural imbalances across healthcare systems with uneven development.

Nationally, the total volume of ACLR procedures between 2017 and 2023 remained markedly lower than that reported in several European and other high-income countries [10,14,15,16]. Comparative analyses across national ligament registries, including those from Sweden, the United Kingdom, New Zealand, and Norway, have reported broad variation in reconstruction rates, ranging from 4.1 to 51.3 per 100,000 inhabitants, as well as substantial differences in treatment delay and surgical practice [33]. In this context, Bucharest–Ilfov lies toward the upper end of the range reported in high-access registry settings, whereas multiple Romanian regions fall at or below the lower bound, suggesting substantial under-provision in parts of the country.

International registry infrastructure is also relevant for interpreting cross-country differences: Scandinavian knee ligament registries were developed specifically to enable systematic surveillance and benchmarking of ACLR practice and outcomes, illustrating how organized service delivery and reporting can shape both measured utilization and quality monitoring [34].

### 4.2. Temporal Dynamics and the COVID-19 Pandemic

The *Year* × *Region* interaction model indicates that the national decline in ACLR incidence during 2020–2021 was not uniform. Economically stronger regions (Centre, West, North-West) showed faster rebounds after 2021, whereas lower-GDP regions (South and South-West) had delayed or minimal recovery. This suggests that the pandemic shock likely amplified pre-existing inequalities: regions with stronger baseline capacity recovered elective surgical volume more rapidly, while resource-limited regions remained constrained.

Although our administrative dataset does not measure pathway mechanisms directly (e.g., waiting times, diagnostic throughput, or rehabilitation access), international evidence strongly supports the plausibility of unequal elective-care recovery after COVID-19 disruptions. Global modelling documented extensive cancellations of elective surgery during peak disruption and emphasized the need for dedicated recovery planning [35]. Subsequent analyses highlighted the growth of elective backlogs as a major post-pandemic challenge [36]. OECD/EU monitoring reported missing volumes of elective procedures in 2020 and worsening waiting times relative to pre-pandemic levels, with recovery remaining incomplete in several systems [37,38,39]. Moreover, empirical evidence from Italy suggests that recovery in surgical volumes may be socially unequal, with disadvantaged strata benefiting more slowly from post-lockdown recovery, consistent with the hypothesis that shocks can widen inequities where baseline capacity is limited [40]. Together, these sources support interpreting the heterogeneous regional rebound observed in Romania as an indicator of differential resilience in elective orthopedic care.

### 4.3. Workforce and Economic Determinants

Multivariable analyses in this study identified regional GDP per capita and orthopedic surgeon density as independent predictors of ACLR incidence, reinforcing the strong relationship between economic development, workforce availability, and access to specialized procedures. This association is consistent with OECD analyses demonstrating that variations in healthcare utilization across regions are correlated with resource availability and economic capacity [30,41].

In essence, wealthier regions with larger specialist workforces performed substantially more knee reconstructions. This finding aligns with broader patterns in the Romanian healthcare system, where more developed regions (e.g., Bucharest–Ilfov, West, Center) tend to attract healthcare resources and personnel, while poorer or predominantly rural regions remain underserved. Nationwide statistics show an extremely skewed distribution of medical professionals: over 90% of doctors in Romania practice in urban areas, leaving rural communities with less than 10% of physicians [21].

The workforce-adjusted model (ACLR volume per available surgeon) indicates that disparities are not explained solely by the number of surgeons. When normalized by workforce, several regions show higher ACLR volume per surgeon compared with Bucharest–Ilfov, which likely reflects differences in case allocation and institutional roles (e.g., the capital’s larger workforce distributed across tertiary activities, teaching, trauma and complex care), as well as within-region centralization to a limited number of high-volume units in non-capital regions. Therefore, workforce density is necessary but not sufficient: functional capacity and organization of care pathways remain critical.

Comparable workforce disparities have been reported in other EU countries [16], but their magnitude may differ by context. While the observed territorial disparities in ACL reconstruction activity are substantial, these findings should not be interpreted as evidence of clinical under- or overuse in the absence of patient-level data. The present analysis reflects patterns of service provision rather than appropriateness of care. Both under-treatment and potential over-treatment are plausible mechanisms underlying the geographic gradient. In low-access regions, limited surgical capacity, workforce shortages, and long travel distances likely contribute to unmet clinical need and delays in definitive treatment. Conversely, in high-access regions such as Bucharest–Ilfov, where diagnostic and operative resources are abundant, some degree of supply-driven utilization cannot be excluded. However, supplier-induced demand cannot be evaluated in the present administrative dataset because clinical indications, injury severity, activity levels, non-operative management, and outcomes are not available.

Similar dual dynamics have been described within the unwarranted variation framework, where regional procedure rates may reflect both access constraints and supply-sensitive utilization related to local availability of resources [42]. Accordingly, the inequalities identified here should be interpreted as structural inequities in service availability and accessibility, not necessarily as differences in clinical indication or appropriateness of intervention.

### 4.4. Role of the Private Sector and Equity Considerations

The role of the private sector in these ACLR deliveries also varied substantially across regions. Bucharest–Ilfov, despite its high overall volume, performed most ACLRs in public hospitals, reflecting the concentration of academic and tertiary orthopedic centers in the capital. Conversely, several other regions relied heavily on private providers, suggesting that private facilities compensate for insufficient public-sector capacity where local demand and economic conditions allow. The multivariable logistic model further supports a supply-driven explanation for private-sector utilization. The proportion of private orthopedic surgeons was the strongest predictor of undergoing ACLR in a private hospital, alongside a positive effect of GDP. The extremely large region-level odds ratios should be interpreted cautiously: they reflect the very low baseline private share in Bucharest–Ilfov (reference) and near-complete private dominance in some regions/years, which can inflate relative odds. Practically, these results indicate a structural segmentation of service delivery across regions rather than individual-level preference alone

These mixed findings suggest that while the public sector remains the main ACLR provider overall, the private sector has become an important player in regions with adequate demand and ability to pay. However, this dynamic introduces potential equity concerns. Private procedures are typically financed out-of-pocket, and access may be restricted for lower-income patients, potentially reinforcing regional and socioeconomic disparities. On the other hand, increased private capacity may also alleviate pressure on the public system and shorten waiting times, particularly in post-pandemic recovery contexts. European analyses on backlog management emphasize the importance of system-wide strategies and coordination of available capacity, while protecting equity of access [39,43].

### 4.5. Interpretation of Inequality Metrics and Sensitivity Analysis

The magnitude of geographic inequality observed in this study is considerable. Several regions exhibited ACLR incidences near zero, while tertiary centers in Bucharest managed disproportionately high volumes. National Gini coefficients exceeded 0.75 in most years. Additional simulations incorporating unreported out-of-pocket activity suggested that missing private-sector data might slightly reduce national inequality estimates; however, even under optimistic assumptions, adjusted Gini coefficients remained above 0.66, confirming that observed disparities reflect genuine differences in service distribution rather than data artifacts. Thus, the inequality values presented likely represent conservative lower bounds of true disparities

International evidence indicates that such disparities are often linked to uneven distribution of medical specialists, concentration of high-capacity hospitals, and regional differences in economic development [41,44]. In Romania, the urban–rural divide in healthcare workforce distribution is pronounced, with medical specialists disproportionately clustered in urban and economically developed areas [45]. Importantly, Romania has no hospitals located in rural localities (communes or villages), meaning that all surgical services, including ACL reconstruction, are delivered exclusively in urban settings. In practice, ACLR is performed almost entirely in large cities and especially in university-based tertiary centers, leaving residents of rural and small-town areas without local access and dependent on substantial travel distances to obtain care.

Overall, inequality metrics consistently demonstrate that Romania exhibits a highly polarized territorial distribution of ACL reconstruction, with access strongly dictated by geographic location and regional socioeconomic development. Taken together, both national and intra-regional metrics reveal persistent, large-scale geographic disparities in ACL reconstruction availability, with surgical activity highly concentrated in a minority of counties and development regions. These patterns reinforce the structural barriers identified in the multivariate models and highlight the need for targeted regional capacity strengthening.

Similarly, this finding is consistent with other domains of health service delivery. For example, a recent geospatial analysis of Romanian emergency care found that approximately 65% of the rural population lives in areas with limited or no access to emergency units, with some remote counties requiring more than 2 h of travel time to reach care [46]. Similar urban–rural inequities in infrastructure and workforce are clearly reflected in our ACLR data. Moreover, insufficient public funding and longstanding under-investment in healthcare likely exacerbate these disparities [21]. Romania’s health expenditure per capita and as a share of GDP remain among the lowest in the EU, contributing to chronic shortages of medical staff and modern facilities in many counties [21]. These systemic weaknesses create a self-reinforcing cycle: specialists cluster in a few centers of excellence, leaving other areas without the personnel or case volumes required to sustain ACL reconstruction services.

Finally, sex-based analyses showed minimal evidence of sex-related inequity in access to ACLR after adjustment. GDP, surgeon supply, and year were not associated with the likelihood of a case being male, and regional differences were largely absent. This supports the conclusion that observed ACLR inequalities in utilization and access opportunity are primarily structural and socioeconomic rather than demographic.

Ultimately, our results support the conclusion that structural disparities, regional economic development, healthcare workforce distribution, and facility availability are the primary determinants of whether patients can access ACL reconstruction. Encouragingly, we did not find evidence that demographic factors such as sex or age biased access, suggesting that when ACLR services are available, they appear to be offered relatively equitably. The key challenge, therefore, is expanding service availability into underserved regions.

Although this study relied on secondary administrative data, multiple steps were taken to ensure the reliability and internal consistency of the findings. In the absence of national arthroscopy registries, the CNAS–INSP discharge datasets represent the only standardized nationwide source of procedural information in Romania. To strengthen methodological robustness, all regression models were re-estimated excluding high-volume outlier regions, and multiple alternative specifications were tested. Additionally, a sensitivity analysis was conducted to simulate unreported out-of-pocket activity in fully private clinics, based on empirical workload assumptions and surgeon distribution. These strategies align with recommended practices in administrative database research and sensitivity analysis, supporting the robustness of both incidence estimates and inequality metrics [47,48].

### 4.6. Advantages and Disadvantages of the Study

This study has several methodological strengths. It uses a comprehensive national administrative dataset covering all insured ACL reconstruction procedures over a seven-year period, ensuring high representativeness and comparability across regions. The integration of surgical, workforce, and economic indicators enables a robust assessment of the structural determinants of access. Furthermore, the multilevel modelling framework, supplemented by temporal interaction and sensitivity analyses, provides strong inferential support for the robustness of the findings.

Despite these strengths, several limitations must be acknowledged. This analysis is based on administrative procedure codes, which may be subject to miscoding or incomplete reporting. As with most administrative datasets, clinical granularity is limited, preventing differentiation between low injury incidence, variation in treatment preferences, and constraints in surgical service availability. Patient mobility also remains unaccounted for. Individuals from low-service counties who travel to higher-volume regions for surgery may inflate local incidence rates in the receiving areas, distorting the geographic distribution of need versus capacity. A further limitation concerns measurement of the surgical workforce. The total number of orthopedic surgeons was used as a proxy for operative capacity, since national data does not distinguish between subspecialties or procedural activity. Not all orthopedic surgeons perform ACLR, and some focus on trauma or general orthopedics. Even so, the strong association between total surgeon supply and ACLR incidence supports its validity as a structural indicator.

Finally, as in all studies using health system administrative data, broader structural and behavioral determinants remain unmeasured. Factors such as referral practices, diagnostic imaging availability, regional sports participation, and postoperative rehabilitation capacity were not captured and may also contribute to observed disparities. Future research should integrate patient-level data and develop a national ligament registry to enable outcome tracking and benchmarking with established registry systems [34].

### 4.7. Practical Implications

The findings of this study have direct relevance for healthcare policy and surgical planning in Romania. Regional inequalities in ACL reconstruction are driven primarily by structural determinants, economic development, workforce distribution, and hospital availability, rather than demographic demand. When interpreted alongside our previous epidemiological analysis of acute ACL ruptures [49], the present findings illustrate the full spectrum of disparities along the ACL care pathway in Romania, from injury occurrence to surgical treatment. Targeted policies are therefore needed to improve regional surgical capacity, redistribute specialists, and strengthen secondary orthopedic centers in underserved regions. Integrating private-sector capacity into coordinated regional referral systems and monitoring elective surgical access through standardized national indicators could enhance both equity and efficiency. These findings provide a foundation for evidence-based planning aimed at strengthening regional resilience and ensuring more uniform access to specialized orthopedic care across the country. Finally, addressing these disparities will require long-term investment in surgical workforce development, infrastructure expansion, and coordinated system-level action. International analyses emphasize that coordinated policy action is crucial to mitigate geographic disparities in healthcare delivery and to ensure more uniform access to essential surgical procedures [41,44].

Addressing these disparities will require long-term investment in surgical workforce development, infrastructure expansion, and system-level coordination to ensure that patients across all Romanian regions have equitable access to modern reconstructive care.

## 5. Conclusions

This nationwide analysis highlights substantial and persistent territorial disparities in access to anterior cruciate ligament reconstruction (ACLR) in Romania. Procedure rates varied widely across regions, with high-volume activity concentrated in Bucharest–Ilfov and minimal availability in several southern regions. These differences were strongly associated with regional economic development, orthopedic workforce distribution, and the uneven presence of public and private surgical infrastructure. Because hospitals, and thus ACLR services, are located exclusively in urban areas, access for residents of rural and economically disadvantaged regions is substantially limited.

Multivariable models confirmed that GDP per capita, surgeon density, and private-sector capacity independently predicted ACLR incidence, underscoring the structural nature of these inequities. Temporal modeling further revealed that the COVID-19 pandemic exacerbated existing disparities, as regions with lower resources experienced delayed recovery in elective surgical activity.

Targeted investment in regional surgical capacity, improved workforce distribution, and development of coordinated referral pathways are needed to ensure equitable access. Reducing these disparities is essential for strengthening musculoskeletal care and improving access to reconstructive surgery nationwide.

## Figures and Tables

**Table 1:** Descriptive statistics of ACL reconstruction rates (per 100,000 population) and male-to-female ratio.

Region	Median	Mean	SD	IQR	Min	Max	Sex Ratio (Median)
Bucharest–Ilfov	40.015	32.633	15.382	16.865	0.180	44.090	2.745
Centre	13.320	14.647	12.431	17.777	0.770	45.830	2.574
North-East	3.330	8.304	10.438	14.320	0.220	28.790	2.500
North-West	3.040	10.253	13.453	8.750	0.470	44.320	2.167
West	6.345	10.149	9.813	16.717	0.490	26.590	2.736
South-East	2.390	2.789	1.705	2.300	0.300	6.840	1.875
South	0.930	2.544	2.996	2.800	0.140	9.890	1.000
South-West	0.670	0.774	0.586	0.510	0.260	2.350	0.000

SD = standard deviation; IQR= interquartile range; Sex ratio = male cases/female cases. The values represent pooled yearly region-level observations (2017–2023).

**Table 2:** Selected Significant Dunn Post Hoc Comparisons.

Comparison	*z*	*ES*	Adjusted *p* (Holm)
Bucharest–Ilfov vs. North-East	3.29	0.676	0.021
Bucharest–Ilfov vs. South	3.95	0.783	0.002
Bucharest–Ilfov vs. South-East	3.33	0.750	0.019
Bucharest–Ilfov vs. South-West	4.95	0.750	<0.001
Centre vs. North-East	3.04	0.403	0.047
Centre vs. South	3.84	0.720	0.003
Centre vs. South-East	3.04	0.608	0.047
Centre vs. South-West	5.16	0.932	<0.001
North-West vs. South	2.40	0.448	0.264 (trend)
North-West vs. South-West	3.77	0.814	0.004
South vs. West	2.23	0.533	0.358 (trend)
South-East vs. South-West	2.38	0.751	0.264 (trend)

*z* = standardized Dunn test statistic; *ES* = effect size. Only statistically significant results (Holm-adjusted *p* < 0.05) and notable trends are reported.

**Table 3:** Descriptive statistics for the proportion of private and public ACL reconstructions.

Region	Median Private	Mean Private	Max Private	Median Public	Mean Public	Min Public
Bucharest–Ilfov	0.033	0.034	0.078	0.967	0.966	0.922
Centre	0.000	0.204	0.835	1.000	0.796	0.165
North-East	0.000	0.077	0.481	1.000	0.923	0.519
North-West	0.000	0.219	0.894	1.000	0.781	0.106
South	0.000	0.044	0.667	1.000	0.956	0.333
South-East	0.000	0.285	1.000	1.000	0.715	0.000
South-West	0.000	0.000	0.000	1.000	1.000	1.000
West	0.028	0.236	0.585	0.972	0.764	0.415

Private proportion = cases performed in private hospitals/total ACL cases; Public proportion = 1 − Private.

**Table 4:** Descriptive Statistics for Socioeconomic and Healthcare Capacity Indicators.

Region	GDP_2021 (€)		Surgeons_per_100 k	Hospitals_ per_100 k	Cases_per_Surgeon
	Median	Mean	Median	Median	Median
Bucharest–Ilfov	23,724	23,724	12.447	0.471	0.465
Centre	11,607	11,483	6.986	0.600	1.009
North-East	7474	7583	3.424	0.220	0.105
North-West	9543	10,812	4.552	0.338	0.321
South	8504	8797	2.671	0.000	0.000
South-East	9017	9775	2.840	0.076	0.050
South-West	9828	9590	5.338	0.000	0.000
West	10,669	11,801	8.081	0.077	0.043

GDP = gross domestic product.

**Table 5:** Multivariate Poisson Regression for ACL Reconstruction Incidence (2017–2023) (*p* value < 0.001 for both models).

Predictor	Main Model		Sensitivity Model (Excluding BI)	
	IRR	95% CI	IRR	95% CI
Year	0.97	0.95–0.98	0.96	0.945–0.971
GDP per capita (*z*-score)	3.99	2.96–5.37	1.96	1.85–2.07
Private-sector proportion	2.94	2.14–4.03	1.60	1.45–1.78
Orthopedic surgeons (count)	1.02	1.018–1.022	1.020	1.019–1.021
North-East	0.77	0.71–0.84	0.77	0.70–0.85
North-West	0.50	0.46–0.53	0.50	0.46–0.54
Centre	0.73	0.69–0.76	0.73	0.69–0.77
South	0.16	0.14–0.19	0.16	0.14–0.19
South-East	0.14	0.12–0.16	0.14	0.12–0.16
South-West	0.05	0.04–0.07	0.05	0.037–0.067
West	0.07	0.06–0.08	0.066	0.058–0.076
Reference region	Bucharest–Ilfov		(excluded)	

BI = Bucharest–Ilfov, IRR = Incidence Rate Ratio, CI = Confidence Interval, Main model includes all NUTS-2 regions with Bucharest–Ilfov as reference. Sensitivity Model excludes all Bucharest–Ilfov data to assess model stability. Both models fitted using robust SEs; dependent variable = annual ACL reconstructions per county.

**Table 6:** Extended Poisson Model for ACL Reconstruction Incidence (2017–2023) with *Year* × *Region* Interaction.

Predictor	IRR	95% CI	*p*-Value
GDP (*z*-score)	1.77	1.67–1.88	<0.001
Orthopedic surgeons (count)	1.02	1.02–1.02	<0.001
Private-sector proportion	1.16	1.03–1.30	0.013
*Centre* × 2019	1.42	1.15–1.75	0.001
*Centre* × 2020	3.17	2.45–4.10	<0.001
*Centre* × 2021	4.22	3.30–5.40	<0.001
*Centre* × 2023	3.52	2.76–4.50	<0.001
*North*-*East* × 2019	2.02	1.61–2.54	<0.001
*North*-*East* × 2020	4.35	3.31–5.70	<0.001
*North*-*East* × 2021	6.68	5.14–8.64	<0.001
*North*-*East* × 2022	1.69	1.36–2.11	<0.001
*North*-*East* × 2023	6.35	5.00–8.09	<0.001
*North*-*West* × 2019	1.63	1.35–1.96	<0.001
*North*-*West* × 2020	2.67	2.10–3.40	<0.001
*North*-*West* × 2021	2.26	1.75–2.90	<0.001
*North*-*West* × 2023	2.61	2.07–3.31	<0.001
*South* × 2018	3.95	1.82–9.83	0.001
*South* × 2019	11.29	5.27–28.00	<0.001
*South* × 2020	10.52	4.08–28.90	<0.001
*South* × 2021	8.17	2.77–24.20	<0.001
*South* × 2022	7.57	3.48–18.90	<0.001
*South* × 2023	22.9	10.4–50.1	<0.001
*South*-*East* × 2019	2.06	1.38–3.06	<0.001
*South*-*East* × 2020	5.70	3.70–8.83	<0.001
*South*-*East* × 2021	4.58	2.89–7.20	<0.001
*South*-*East* × 2023	2.14	1.35–3.37	0.001
*South*-*West* × 2020	18.4	6.08–67.70	<0.001
*South*-*West* × 2021	8.24	1.13–42.80	0.016
*South*-*West* × 2022	4.05	1.06–16.45	0.038
*South*-*West* × 2023	17.74	6.27–63.10	<0.001
*West* × 2018	1.46	1.04–2.07	0.032
*West* × 2019	2.81	2.02–3.94	<0.001
*West* × 2020	5.37	3.75–7.75	<0.001
*West* × 2021	5.54	3.97–7.81	<0.001
*West* × 2023	5.65	4.10–7.80	<0.001

IRR = incidence rate ratio; CI = confidence interval; GDP = gross domestic product. Reference: Bucharest–Ilfov in 2017 (baseline year. Only statistically significant interaction terms (*p* < 0.05) are shown.

**Table 7:** Workforce-adjusted Poisson regression.

Predictor	Estimate	IRR	95% CI	*p*
*z*-scored GDP	0.576	1.78	1.70–1.86	<0.001
Year	–0.032	0.97	0.96–0.98	<0.001
Centre	2.680	14.6	11.7–18.2	<0.001
North-East	3.050	21.2	16.5–26.9	<0.001
North-West	2.460	11.7	9.7–14.1	<0.001
South	2.167	8.7	6.6–11.6	<0.001
South-East	1.406	4.1	3.2–5.2	<0.001
South-West	0.306	1.36	0.92–1.96	0.135
West	1.504	4.5	3.7–5.5	<0.001

IRR = incidence rate ratio; CI = confidence interval; GDP = gross domestic product. offset = log(Total_surgeons). Reference region: Bucharest–Ilfov.

**Table 8:** Binomial Logistic Regression Predicting Private-Sector ACL Reconstruction.

Predictor	OR	95% CI	*p*
Year	0.97	0.94–1.00	0.044
GDP (*z*-score)	1.58	1.38–1.82	<0.001
Total surgeons	1.01	1.01–1.02	<0.001
Private surgeons (%)	21.03	10.81–41.25	<0.001
Centre	23,818	8924–65,545	<0.001
North-East	19,187	3409–51,756	<0.001
North-West	8579	3409–22,301	<0.001
South	766	97–4050	<0.001
South-East	60,447	21,219–178,375	<0.001
South-West	0.002	—	0.987
West	1600	777–3389	<0.001

OR = odds ratio, CI = confidence interval. Reference: Bucharest–Ilfov.

**Table 9:** Sex-Based Logistic Regression Predicting Male Sex.

Predictor	OR	95% CI	*p*
GDP (*z*-score)	1.10	0.98–1.23	0.122
Total surgeons	1.00	0.998–1.002	0.965
Year	0.99	0.97–1.02	0.508
Centre	1.57	0.84–2.94	0.159
North-East	2.00	1.05–3.81	0.033
North-West	1.59	0.92–2.75	0.097
South	0.79	0.27–2.28	0.605
South-East	1.82	0.95–3.45	0.069
South-West	1.70	0.91–3.15	0.096
West	1.37	0.83–2.25	0.218

OR = odds ratio, CI = confidence interval. Reference: Bucharest–Ilfov.

**Table 10:** National Inequality Metrics for ACL Reconstruction Incidence.

Year	Gini Coefficient	Gini Adjusted (Sensitivity Analyses)			P90/P10 Ratio *
		Low	Medium	High	
2017	0.842	0.738	0.718	0.713	1.88 × 10^10^
2018	0.822	0.713	0.691	0.684	1.95 × 10^10^
2019	0.806	0.694	0.673	0.668	2.17 × 10^10^
2020	0.777	0.688	0.688	0.692	1.43 × 10^10^
2021	0.774	0.682	0.682	0.685	1.69 × 10^10^
2022	0.752	0.665	0.662	0.665	2.46 × 10^10^
2023	0.767	0.669	0.667	0.671	2.58 × 10^10^

* P90/P10 ratio remained >10^9^ in all years and scenarios.

**Table 11:** Internal Inequality by Development Region (NUTS-2).

Region	Gini Coefficient	Gini Adjusted (Sensitivity Analyses)		
		Low	Medium	High
Bucharest–Ilfov	0.555	0.290	0.223	0.191
Centre	0.566	0.546	0.567	0.579
Nord-Est	0.764	0.648	0.604	0.576
Nord-Vest	0.736	0.591	0.589	0.600
Sud	0.867	0.744	0.740	0.747
Sud-Est	0.668	0.662	0.688	0.701
Sud-Vest	0.761	0.438	0.448	0.451
Vest	0.756	0.537	0.518	0.511

## Data Availability

Data are contained in the manuscript. More information should be requested from the first author.

## Abbreviations

The following abbreviations are used in this manuscript:

ACL	Anterior Cruciate Ligament
ACLR	Anterior Cruciate Ligament Reconstruction
BI	Bucharest–Ilfov
CNAS	Casa Națională de Asigurări de Sănătate (Romanian National Health Insurance House)
DRG	Diagnosis-Related Group
GDP	Gross Domestic Product
INSSE	Institutul Național de Statistică (National Institute of Statistics)
INSP	Institutul Național de Sănătate Publică (National Institute of Public Health)
NCSP	Nordic Medico-Statistical Committee Classification of Surgical Procedures
NUTS-2	Nomenclature of Territorial Units for Statistics Level 2
